# Author Correction: Comparative mode of action of the antimicrobial peptide melimine and its derivative Mel4 against *Pseudomonas aeruginosa*

**DOI:** 10.1038/s41598-019-49307-6

**Published:** 2019-09-10

**Authors:** Muhammad Yasir, Debarun Dutta, Mark D. P. Willcox

**Affiliations:** School of Optometry and Vision Science, University of New South Wales, Sydney, Australia

Correction to: *Scientific Reports* 10.1038/s41598-019-42440-2, published online 08 May 2019

This Article contains a typographical error in the Results section under subheading “Interaction with Lipopolysaccharides” where,

“The LAL test was used to assess the ability of the AMPs to interact and neutralize the LPS. Both melimine and Mel4 inhibited LPS’s ability to stimulate amoebocyte lysate in a dose-dependent manner as demonstrated in Table 3.”

should read:

“The LAL test was used to assess the ability of the AMPs to interact and neutralize the LPS. Both melimine and Mel4 inhibited LPS’s ability to stimulate amoebocyte lysate in a dose-dependent manner as demonstrated in Table 2.”

