# Supplementary material for: Comparative mode of action of the antimicrobial peptide melimine and its derivative Mel4 against Pseudomonas aeruginosa
Source: Sci Rep. 2019 May 8;9:7063. doi: 10.1038/s41598-019-42440-2 (PMC6506473; doi:10.1038/s41598-019-42440-2)
Supplement: Supplementary file 1 — Table S1 [file 41598_2019_42440_MOESM1_ESM.pdf]

Title: Comparative mode of action of the antimicrobial peptide melimine and its derivative Mel4 against *Pseudomonas aeruginosa*

Muhammad Yasir, Debarun Dutta, Mark DP Willcox

School of Optometry and Vision Science, University of New South Wales, Sydney, Australia

Table S1. Increase in fluorescence units due to release of DiSc3-5 after membrane depolarization. Data presented as means (±SD).

Time (min)	*P. aeruginosa* 6206						*P. aeruginosa* Paer1						*P. aeruginosa* ATCC 19660					
	Melimine		Mel4		DMSO 20%	Buffer	Melimine		Mel4		DMSO 20%	Buffer	Melimine		Mel4		DMSO 20%	Buffer
	1x	2x	1x	2x			1x	2x	1x	2x			1x	2x	1x	2x		
30	41±1	44±2	31±1	39±1	44±1	0±0	37±3	37±2	27±2	30±2	39±1	0±0	39±1	41±2	29±1	30±2	44±4	0±0
60	52±2	59±1	43±3	47±1	55±1	0±0	50±1	51±1	37±3	43±2	54±1	0±0	50±1	58±1	41±3	43±2	53±2	0±0
90	62±2	66±2	58±3	63±2	68±3	0±0	61±3	63±1	46±3	54±1	65±0	0±0	60±1	64±3	48±3	50±2	67±2	0±0
120	66±1	76±2	69±1	73±1	85±2	1±1	70±1	70±1	52±3	61±2	83±0	0±0	65±1	73±2	57±2	60±2	85±1	0±0
150	74±4	83±3	78±2	81±1	92±2	1±1	79±2	81±12	60±1	68±3	90±0	0±0	72±3	81±2	67±3	69±1	92±2	0±0
180	79±2	84±3	79±2	84±1	94±2	1±1	81±1	81±2	66±2	75±3	92±0	0±0	76±2	83±2	73±3	76±2	94±2	0±0
210	82±2	90±1	80±2	83±2	96±3	1±1	81±2	83±2	69±1	75±3	93±0	0±0	81±3	89±2	74±3	78±2	95±3	1±1
240	84±1	95±2	80±1	83±3	97±3	1±1	82±2	89±2	69±1	75±3	94±0	0±0	82±3	93±1	75±5	79±2	98±2	1±1
270	86±1	99±1	81±1	84±3	99±1	1±1	83±2	89±2	69±1	75±3	99±0	0±0	85±1	98±3	75±3	79±3	100±1	1±1
300	88±2	106±1	81±0	84±4	104±1	1±1	83±1	91±2	69±1	75±3	100±1	0±0	86±2	103±1	75±3	80±4	104±1	1±1

Table S2. Corresponding decrease in viable bacteria due to membrane depolarization (CFU/ml). Data presented as means (±SD).

Time (min)	***P. aeruginosa* 6206**					***P. aeruginosa* Paer1**					***P. aeruginosa* ATCC 19660**				
	**Melimine**		**Mel4**		**Buffe r**	**Melimine**		**Mel4**		**Buffer**	**Melimine**		**Mel4**		**Buffer**
	**1x**	**2x**	**1x**	**2x**		**1x**	**2x**	**1x**	**2x**		1x	2x	1x	2x	
30	804767/404	506000/1732	6019333/33486	6032667/56580	10000000/0	805333/577	506333/2309	6022000/38105	502000/3464	10000000/0	7617667/2540	505533/924	6022333/38682	6015000/25981	10000000/0
60	653000/5169	138333/2887	5024667/421724	4520000/343641	10000000/0	806333/2309	322667/4619	3520000/34641	352500/4330	10000000/0	4011000/3868	132000/3464	5025667/44456	4521852/37848	10000000/0
90	4013332309	86963/6047	1015000/25981	3902233/3868	10000000/0	652933/5081	102467/4272	1902767/4792	189233/8875	10000000/0	2001500/1963	71100/1905	1007333/12702	993000/4359	10000000/0
120	202000/3464	51400/1277	881867/3233	801867/3233	10000000/0	403133/5427	81033/1002	1014667/25403	111000/19053	10000000/0	1015000/4503	45133/231	877000/5196	802800/4850	10000000/0
150	101600/2117	31333/577	551500/2598	501767/3060	10000000/0	219133/11325	50733/1270	686667/11547	85000/1000	10000000/0	802533/4179	18267/252	551667/2887	501833/3175	10000000/0
180	61200/1058	17833/289	370147/10003	360800/1386	10000000/0	102167/3753	40667/1155	227667/2517	73333/2887	10000000/0	301500/4258	13033/1674	381633/2829	363100/5369	10000000/0
210	31333/2309	10900/361	93481/4413	81333/1155	10000000/0	63033/3406	32433/4215	101133/1963	45000/1000	10000000/0	81067/173	10733/635	107667/13279	249700/43798	10000000/0
240	10717/625	7017/475	45600/6031	39367/679	10000000/0	35767/1328	25100/173	40333/577	36667/2887	10000000/0	52667/1206	7833/289	76333/289	101067/1361	10000000/0
270	4433/513	2233/252	23698/711	21198/1951	10000000/0	26067/1848	16333/2309	22100/2007	22333/2517	10000000/0	25333/2598	4300/265	52333/2082	48833/2021	10000000/0
300	922/84	660/10	8217/1190	4918/84	10000000/0	13222/1170	14542/3288	14778/1251	15144/3552	10000000/0	9233/1232	833/153	24833/4752	15855/2023	10000000/0

Table S3. Increase in relative fluorescence units due to interaction of Sytox Green Dye with DNA. Data presented as means (±SD).

Time (min)	*P. aeruginosa* 6206						*P. aeruginosa* Paer1						*P. aeruginosa* ATCC 19660					
	Melimine		Mel4		Triton -X 1%	Buffer	Melimine		Mel4		Triton -X 1%	Buffer	Melimine		Mel4		Triton –X 1%	Buffer
	1x	2x	1x	2x			1x	2x	1x	2x			1x	2x	1x	2x		
5	8±3	11±2	4±2	6±01	21±2	1±0	5±1	6±1	5±2	5±1	16±3	1±0	14±2	15±1	8±1	7±1	22±0	1±0
10	10±1	11±2	6±1	8±1	32±2	1±0	10±1	10±1	10±1	10±1	15±1	1±0	22±3	24±1	12±7	7±2	23±2	1±0
15	11±2	12±1	7±1	7±2	41±3	1±0	12±2	14±1	11±2	11±1	15±4	1±0	41±5	42±2	13±6	10±5	48±3	1±0
20	12±0	12±0	7±2	9±2	53±3	1±0	22±3	23±0	13±1	14±1	24±5	1±0	45±4	47±2	15±8	11±3	81±6	1±0
25	13±2	17±2	11±2	15±1	64±2	1±0	35±3	33±2	15±0	16±1	32±6	1±0	48±6	54±2	17±8	14±5	107±0	1±0
30	14±4	18±2	10±1	12±2	71±1	1±0	45±4	45±2	25±1	26±1	41±7	1±0	53±5	56±3	19±9	15±5	124±2	1±0
150	35±3	38±1	19±2	18±2	135±8	1±0	91±9	97±3	33±2	32±2	94±8	1±0	67±7	69±4	44±3	46±3	168±12	1±0

Table S4. Percentage (%) increase in extracellular ATP. Data presented as means (±SD).

Time (min)	*P. aeruginosa* 6206					*P. aeruginosa* Paer1					*P. aeruginosa* ATCC 19660				
	Melimine		Mel4		Buffer	Melimine		Mel4		Buffer	Melimine		Mel4		Buffer
	1x	2x	1x	2x		1x	2x	1x	2x		1x	2x	1x	2x	
**0**	0±0	0±0	0±0	0±0	0±0	0±0	0±0	0±0	0±0	0±0	0±0	0±0	0±0	0±0	0±0
**2**	84±2	86±1	37±2	41±1	4±1	75±1	81±2	53±2	54±2	4±0	66±2	69±1	19±2	20±2	3±1
**4**	90±1	90±1	41±1	45±2	4±1	79±1	86±1	57±1	59±1	4±1	70±1	72±1	24±1	25±2	3±1
**6**	92±2	92±1	43±1	47±2	4±1	83±3	90±1	61±2	63±1	4±1	72±2	74±1	25±3	30±2	3±1
**8**	94±1	94±1	44±1	49±1	4±1	90±1	92±1	61±1	65±1	4±0	76±1	79±1	30±2	34±1	4±1
**10**	94±0	95±1	46±1	52±1	4±1	90±1	93±1	63±0	66±2	4±0	79±0	82±1	31±1	35±2	4±1

Table S5. Corresponding decrease in viable bacteria due to release of ATP (CFU/ml). Data presented as means (±SD).

Time (min)	*P. aeruginosa* 6206					*P. aeruginosa* Paer1					*P. aeruginosa* ATCC 19660				
	Melimine		Mel4		Buffer	Melimine		Mel4		Buffer	Melimine		Mel4		Buffer
	1x	2x	1x	2x		1x	2x	1x	2x		1x	2x	1x	2x	
**0**	10000000/0	10000000/0	10000000/0	10000000/0	10000000/0	10000000/0	10000000/0	10000000/00	10000000/0	10000000/0	10000000/0	10000000/0	10000000/0	10000000/0	10000000/0
**2**	10/1	11/1	124556/3344	121578/7099	10000000/0	6167/1850	5767/666	184222/21644	176311/13057	10000000/0	4446/222	3489/317	145267/462	124889/14084	10000000/0
**4**	10/1	11/1	124556/3428	121944/6852	10000000/0	5767/1429	5633/569	183222/20029	176344/13112	10000000/0	4433/2065	3456/269	144333/1155	124556/13623	10000000/0
**6**	10/1	10/1	125489/3455	121978/7231	10000000/0	5700/1539	5600/529	182556/18963	176244/12948	10000000/0	4400/2007	3422/227	145233/404	133889/28202	10000000/0
**8**	10/1	11/1	124789/3455	121578/6880	10000000/0	6167/1850	5633/569	184222/21644	176311/13057	10000000/0	4367/1950	3389/192	144333/1155	123889/12729	10000000/0
**10**	10/1	10/1	124489/2110	121811/7033	10000000/0	5767/1429	5500/436	183222/20029	173011/15342	10000000/0	4333/1893	3356/171	144000/1732	124189/13127	10000000/0

Table S6. Proportional increase of 260 nm absorbing materials (DNA/RNA) at 1x and 2x MIC of peptides. Data presented as means (±SD).

Time (min)	*P. aeruginosa* 6206					*P. aeruginosa* Paer1					*P. aeruginosa* ATCC 19660				
	Melimine		Mel4		Buffer	Melimine		Mel4		Buffer	Melimine		Mel4		Buffer
	1x	2x	1x	2x		1x	2x	1x	2x		1x	2x	1x	2x	
2	6.1±0.1	9.2±0.8	3.3±0.5	3.3±0.7	1.1±0.2	6.4±0.3	9.7±0.4	3.2±0.3	5.4±0.3	1.0±0.0	9.7±0.6	13.1±0.7	4.7±0.6	5.4±0.7	1.1±0.1
**5**	6.1±0.1	9.2±0.8	3.3±0.5	3.3±0.7	1.1±0.2	6.4±0.3	9.7±0.4	3.2±0.3	5.4±0.3	1.0±0.0	9.7±0.6	13.1±0.7	4.7±0.6	5.4±0.7	1.1±0.1
**10**	6.2±0.2	9.8±0.6	3.4±0.4	3.5±0.3	1.2±0.2	6.6±0.3	10.0±0.3	3.3±0.3	5.5±0.4	1.0±0.1	9.8±0.8	13.2±0.7	5.2±0.1	5.6±0.6	1.1±0.1
**15**	6.2±0.2	9.9±0.2	3.4±0.4	3.8±0.1	1.2±0.2	6.6±0.3	10.0±0.1	3.4±0.4	5.5±0.5	1.1±0.1	10.2±1.0	13.3±0.7	5.3±0.2	5.7±0.6	1.1±0.1
**20**	6.6±0.2	10.0±0.3	3.6±0.3	3.7±0.2	1.1±0.2	6.7±0.3	10.1±0.2	3.6±0.4	5.4±0.9	1.1±0.2	10.2±1.0	13.3±0.7	5.1±0.7	5.9±0.7	1.1±0.1
**25**	6.6±0.3	10.1±0.3	3.7±0.2	3.8±0.1	1.1±0.1	6.7±0.3	10.1±0.3	3.9±0.2	5.3±1.5	1.0±0.0	10.2±1.0	13.6±1.2	5.5±0.2	6.2±0.5	1.1±0.1
**30**	6.7±0.2	10.0±0.6	3.7±0.4	3.8±0.2	1.1±0.1	6.8±0.2	10.2±0.3	4.1±0.2	5.4±1.0	1.0±0.1	10.2±1.0	13.6±1.0	5.6±0.3	6.3±0.5	1.1±0.1
**60**	6.7±0.2	10.1±0.6	3.9±0.2	3.9±0.1	1.0±0.0	6.9±0.2	10.3±0.3	4.1±0.3	5.5±1.2	1.0±0.0	10.3±0.9	14.7±0.2	5.7±0.3	6.4±0.5	1.1±0.1
**150**	10±0.5	14±0.7	5.7±0.6	9.4±0.1	1.0±0.0	10.6±0.8	12.8±1.4	6.8±0.3	7.0±0.4	1.0±0.0	12.0±1.0	15.6±0.7	7.7±1.0	7.8±0.9	1.1±0.1

Table S7. Remining OD_{620nm} in percentage (%) following bacterial-lysis by peptides at different time interval. Data presented as means (±SD).

Time (h)	*P. aeruginosa* 6206					*P. aeruginosa* Paer1					*P. aeruginosa* ATCC 19660				
	Melimine		Mel4		Buffer	Melimine		Mel4		Buffer	Melimine		Mel4		Buffer
	1x	2x	1x	2x		1x	2x	1x	2x		1x	2x	1x	2x	
**0**	100±0	100±0	100±0	100±0	100±0	100±0	100±0	100±0	100±0	100±0	100±0	100±0	100±0	100±0	100±0
**2**	77±4	82±6	86±3	78±11	100±0	90±2	79±8	76±4	65±9	92±3	78±5	75±6	93±5	86±6	100±0
**6.5**	70±5	76 ±5	80±4	70±8	99±1	67±5	56±4	59±4	52±7	88±3	63±6	60±4	76±3	60±4	98±1
**24**	58±4	60±5	68±8	55±6	100±1	43±2	37±3	23±4	8±2	87±4	ND	ND	ND	ND	ND

ND= not determined
